# Microcystic adnexal carcinoma (MAC)-like squamous cell carcinoma as a differential diagnosis to Bell´s palsy: review of guidelines for refractory facial nerve palsy

**DOI:** 10.1186/s40463-016-0180-0

**Published:** 2017-01-05

**Authors:** S. K. Mueller, H. Iro, M. Lell, F. Seifert, C. Bohr, C. Scherl, A. Agaimy, M. Traxdorf

**Affiliations:** 1 Department of Otolaryngology, Friedrich-Alexander University Erlangen-Nürnberg, Waldstrasse 1, Erlangen, 91054 Germany; 2 Department of Radiology, Klinikum Nuremberg, Nuremberg, Germany; 3 Department of Neurology, Friedrich-Alexander University Erlangen-Nürnberg, Schwabachanlage 6, Erlangen, 91054 Germany; 4 Department of Pathology, Friedrich-Alexander University Erlangen-Nürnberg, Krankenhausstraße 8-10, Erlangen, 91054 Germany

**Keywords:** Head and neck oncology, Facial nerve palsy, Cranial nerve resection

## Abstract

**Background:**

Bell´s palsy is the most common cause of facial paralysis worldwide and the most common disorder of the cranial nerves. It is a diagnosis of exclusion, accounting for 60–75% of all acquired peripheral facial nerve palsies. Our case shows the first case of a microcystic adnexal carcinoma-like squamous cell carcinoma as a cause of facial nerve palsy.

**Case presentation:**

The patient, a 70-year-old Caucasian male, experienced subsequent functional impairment of the trigeminal and the glossopharyngeal nerve about 1½ years after refractory facial nerve palsy.

An extensive clinical work-up and tissue biopsy of the surrounding parotid gland tissue was not able to determine the cause of the paralysis. Primary infiltration of the facial nerve with subsequent spreading to the trigeminal and glossopharyngeal nerve via neuroanastomoses was suspected. After discussing options with the patient, the main stem of the facial nerve was resected to ascertain the diagnosis of MAC-like squamous cell carcinoma, and radiochemotherapy was subsequently started.

**Conclusion:**

This case report shows that even rare neoplastic etiologies should be considered as a cause of refractory facial nerve palsy and that it is necessary to perform an extended diagnostic work-up to ascertain the diagnosis. This includes high-resolution MRI imaging and, as perilesional parotid biopsies might be inadequate for rare cases like ours, consideration of a direct nerve biopsy to establish the right diagnosis.

## Background

Facial nerve paralysis (FNP) involves the paralysis of any structures innervated by the facial nerve, occurring either in infranuclear/nuclear (“peripheral”) or supranuclear (“central”) form. Individuals of all races and ages are affected and can experience significant functional, social and psychological consequences [1]. FNP is a frequent problem, with an incidence of 17 to 35 cases per 100,000 individuals per year [1]. Other etiologies include viral or bacterial infections, autoimmune diseases, malformations, malignancies and traumas [2].

Bell´s palsy (BP; idiopathic facial nerve paralysis) is the most common cause of facial paralysis worldwide and the most common disorder of the cranial nerves [3]. With progressing age, 7–40 patients per year and per 100,000 individuals are affected by BP, with equal gender distribution. BP is a diagnosis of exclusion, accounting for 60–75% of all acquired peripheral FNPs [2].

The generally recommended work-up for new-onset FNP consists of the clinical history, physical examination and regular patient follow-up. Medical therapy with corticosteroids should be started within the first 72 h after the onset of symptoms in patients over the age of 16 with all degrees of severity [3, 4]. This increases the rate of facial nerve recovery [1] and significantly reduces the risk of synkinesis [5]. In patients with severe to complete paresis, the combination of corticosteroids and antivirals can be used [3, 4]. Electrostimulation, routine laboratory testing and surgical decompression is not recommended in non refractory cases within the first 3 months [3, 4]. Eye protection, e.g. wearing a moisture chamber at night, should be applied in every patient with incomplete eye closure, and in severe cases an ophthalmologist should be consulted to avoid cornea damage due to dry eyes [3, 4].

The prognosis for BP is good, with about 70% of the cases resulting in spontaneous, complete recovery. In patients with incomplete paresis, the recovery rate is even higher at 93–98%. Remission starts about 3–4 weeks after onset. Symptoms normally resolve completely within 3–5 months [2, 4]. Although the prognosis for BP is good, not every FNP resolves. Six months after the onset of symptoms at the latest, other differential diagnoses have to be considered in refractory cases [2].

In the following, we describe a complex case of persistent House-Brackmann VI FNP where the diagnosis could only be made after excluding various differential diagnoses.

## Case presentation

A 70-year-old male first presented at the Department of Otorhinolaryngology, Head and Neck Surgery, University of Erlangen-Nürnberg, in 2014 with refractory facial paralysis (House Brackmann VI) that had affected the right side for 2 months. No change in facial nerve function could be seen after intravenous therapy with cortisone [6], ceftriaxone and valacyclovir had been carried out externally for 10 days.

The previous medical history included canal wall down tympanoplasty of the right ear in 1966 due to cholesteatoma. Other symptoms, such as change in hearing, vertigo or tinnitus, were negated.

Clinical examination showed normal otoscopic findings after canal wall down tympanoplasty on the right side. The audiogram showed a severe combined hearing loss with a conductive component of 15 dB on the right side (unchanged during the course), while the left side was normal.

Laboratory examination was unremarkable, including the serology for Lyme´s disease and varicella zoster virus. An ultrasound scan of the parotid gland produced normal findings as well. Metabolic disorders such as diabetes were excluded and a routine ophthalmologic examination was also normal.

The facial nerve electromyogram, performed 3 months after the onset of symptoms, showed no signs of degeneration or reinnervation.

An MRI scan was performed, and recurrent cholesteatoma on the right side was suggested. Additionally, the facial nerve demonstrated contrast enhancement and thickening proximal to the geniculate ganglion up to the peripheral nerve endings; neuritis was assumed. No signs of tumors of the cerebellopontine angle or other intracranial tumors could be detected.

As the routine work-up did not show the cause of the FNP, other differentials had to be considered. Due to the suspicion of recurrent cholesteatoma, surgical exploration of the right mastoid was performed and a small cholesteatoma was removed. Surprisingly, the bony facial canal was intact and no contact with the facial nerve could be seen. It was concluded that this finding could not be regarded as the cause of the FNP. Facial nerve decompression was not performed due to the long segment enhancement of the facial nerve.

Recommended follow-up appointments were not kept for 1 year. At his next visit 1 year after onset of symptoms, the patient complained of a new onset of hypesthesia on the right side of the face in the trigeminal supply territory (V2 + 3).

As the patient had swallowing problems, the renewed clinical examination showed an impaired gag reflex. Contrast swallow showed an insufficiency of the upper esophageal sphincter. Due to this finding and the backdrop phenomenon, an affection of the glossopharyngeal nerve was suspected. As transnasal fiberoptic examination showed aspiration the patient received no more oral nutrition from this point on, and a percutaneous endoscopic gastrostomy tube was placed.

Extensive neurological examinations (gross examination, somatosensory-evoked potentials (SEPs), electroencephalography (EEG), Doppler ultrasound, lumbar puncture, autoimmune testing, plasmapheresis) were carried out to exclude further differentials, but still no diagnosis could be found.

**Fig. 1:**
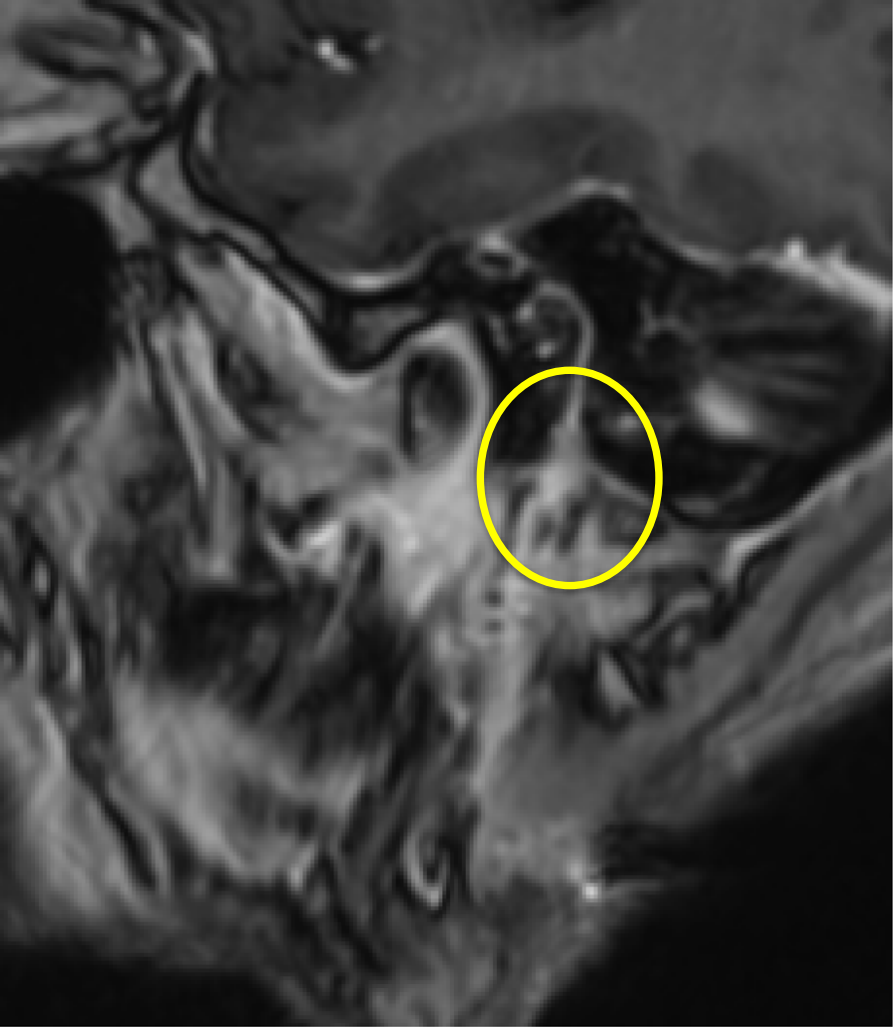
MR-RAGE after contrast (Gd), oblique sagittal: Course of the facial nerve and thickened facial nerve entering the parotid gland

**Fig. 2:**
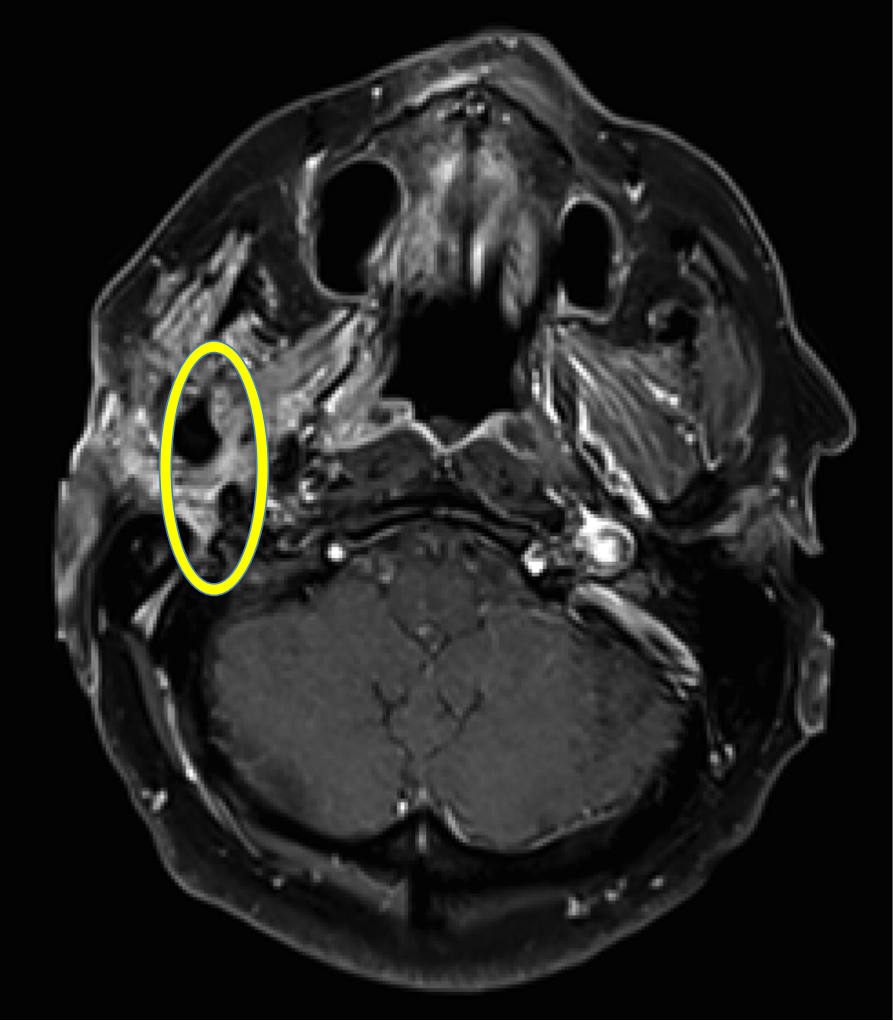
T1 TSE FS after contrast, axial: thickened facial nerve

MRI was repeated, demonstrating abnormal contrast enhancement of the facial and trigeminal nerve and a diffuse contrast-enhancing lesion inside the right parotid gland (Figs. 1 and 2). Explorative surgery of the parotid gland was performed and multiple biopsies were taken from the corresponding areas. Histologically, the biopsies showed no signs of malignancy or inflammation.

After discussing options with the patient, the main stem of the facial nerve was resected.

**Fig. 3:**
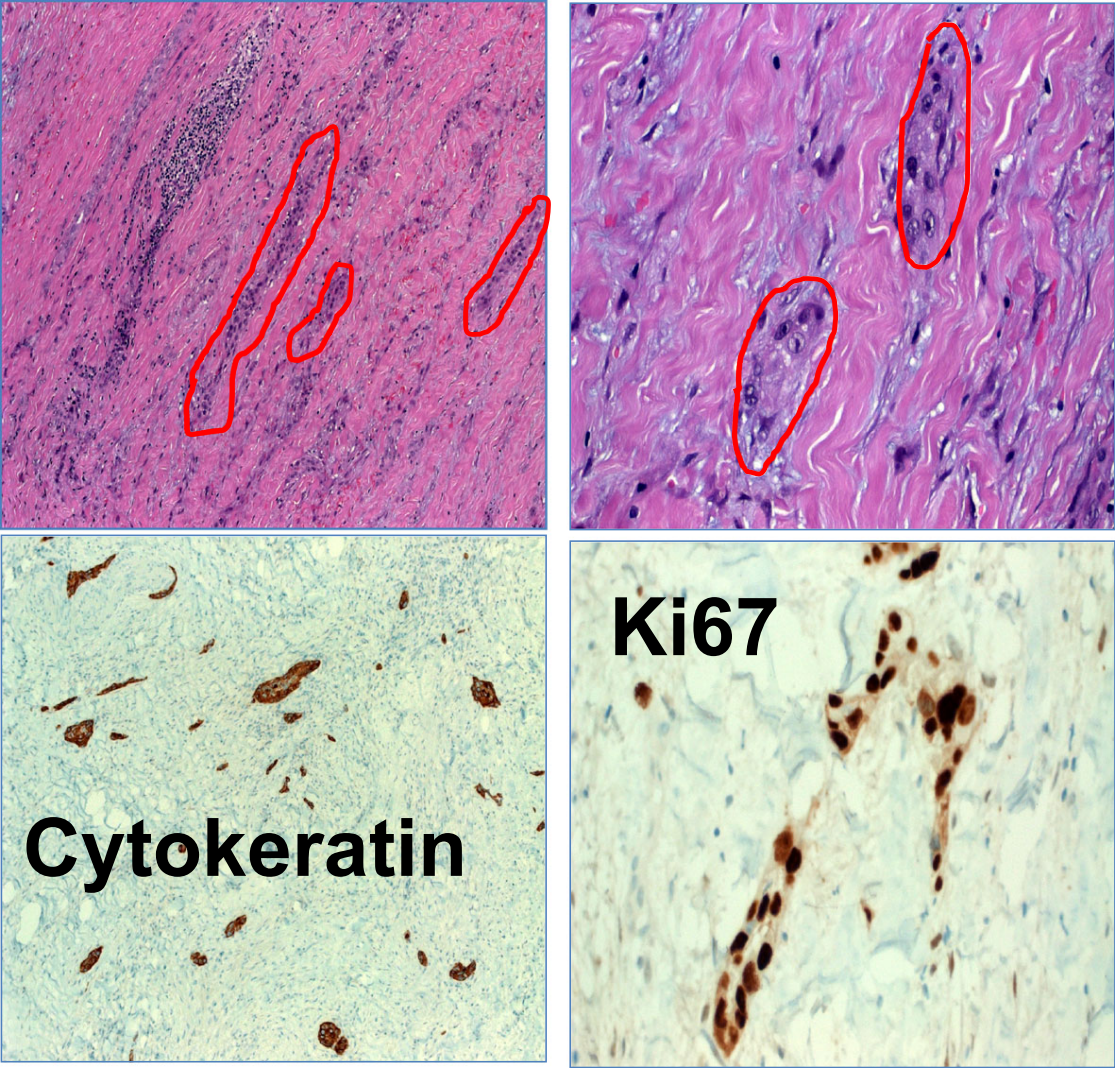
Histological sections. *Top left*: The biopsy of the facial nerve shows scanty, retiform-arranged infiltrates of the carcinoma within abundant inflammatory fibrous tissue. *Top right*: High magnification of tumor infiltrates. *Bottom left*: The CK5 stain shows numerous unexpected tumor nests with small strands and adenoid nests (MAC-like). *Bottom right*: KI67 shows an extremely high proliferation of the tumor cells

Histologically, the facial nerve was found to be infiltrated by squamous cell carcinoma growing in minute nests and cords very reminiscent of microcystic adnexal carcinoma (MAC) of the skin, but with pure squamoid differentiation (Fig. 3). A primary infiltration of the facial nerve with subsequent spreading to the trigeminal and glossopharyngeal nerve via neuroanastomoses was suspected.

An 18-Fluoro-deoxyglucose-Positron-Emission-Tomography/Computed Tomography (PET-CT) showed no new focus, signs of metastases or any other primary tumor.

As surgical treatment was not an option due to the perineural growth of the tumor, radiochemotherapy was recommended at our interdisciplinary tumor conference and was realized (up to a total dose of 66.6 Gy); chemotherapy was also given with CDDP (Cisplatin)).

## Discussion

Microcystic adnexal carcinoma (MAC) is a rare tumor that was first described in 1982 [7]. It is a slow-growing, locally aggressive, deeply infiltrating tumor that can extend into muscles, fat and bones [8]. Perineural or intraneural involvement is characteristic. Although it only rarely metastasizes, the local recurrence rate is high [8]. MAC occurs mainly in the centrofacial skin. However, rare examples have been described in the orbita, the external ear canal, the minor salivary glands, the vulva and the axilla [7]. To date, only six cases have been reported in non-cutaneous head and neck sites, including the tongue (*n* = 3), and one case each in the nasopharynx, floor of the mouth and parotid gland [9].

Histologically, it is characterized by an infiltrative pattern of slightly atypical cells forming cords, nests and a duct-like structure with a prominent desmoplastic stromal reaction [8].

This is the first described case of MAC occurring at the facial nerve (and equiprobably to the other caudal cranial nerves affected) and is a new differential diagnosis for persistent FNP/ BP. Although no clear-cut primary tumor mass could be identified, it is likely that the primary tumor originated within the parotid gland and that the extensive neurotropic growth via nerve anastomoses might have precluded the formation of a larger tumor mass.

Unlike central FNP, in peripheral idiopathic FNPs imaging is not crucial for work-up or treatment in patients with typical clinical findings, as most patients recover within 3–6 months of onset [10]. Imaging is, therefore, only recommended to rule out neoplasms and other causes in patients with no response to initial treatment after 3 months or with progressive facial nerve paralysis [3, 4]. Some authors suggest performing native and contrast-enhanced imaging of the parotid gland, temporal bone and brain if restoration has not occurred within 3 months and a repeat of the imaging after 7 months if a definable cause still cannot be found. In patients with local progression/ recurrence, a second paralysis on the same side, paralysis of isolated branches of the facial nerve or involvement of other cranial nerves, contrast-enhanced imaging should be considered immediately [1, 4, 10]. In our case, imaging was performed within the first 3 months of onset of symptoms to rule out recurrent cholesteatoma.

Close follow-up is important to pick up novel neurologic or ocular findings or in the case of incomplete recovery 3 months after onset. Referrals to other specialties should be performed accordingly [2].

If a thorough diagnostic work-up still does not lead to a diagnosis, other means should be considered. Currently, there are no guidelines concerning soft tissue or facial nerve biopsies. Some authors do, however, suggest considering a biopsy of the surrounding tissue of the facial nerve, e.g. parotid gland tissue in the case of negative imaging at 7 months [1, 5]. In patients with non-resolving FNP, an exploration, or even a biopsy of the facial nerve itself, can be discussed. Factors underlining the need for exploration are pain or regional skin cancer and the involvement of other cranial nerves [11]. In our case, facial and trigeminal nerve biopsies were discussed to force a diagnosis. Due to the long-persisting FNP, the disease progression and the age of the patient, we decided to biopsy the main stem of the facial nerve.

In patients with contrast enhancement of the facial nerve or the surrounding tissue on MRI, the surrounding tissue/ the facial nerve should be biopsied closest to the enhancement. However, it is crucial to know that contrast enhancement of the facial nerve can be seen up to the geniculate ganglion, even in non-diseased patients. The tympanic and mastoid segments do not usually demonstrate enhancement except in cases of neuritis, tumor or palsy. Asymmetric enhancement between the ipsi- and contralateral sides is highly suggestive, although not very specific. Contrast enhancement beyond the stylomastoid notch as well as thickening of the nerve should raise the suspicion of inflammatory or neoplastic involvement.

An MRI scan can also be of prognostic value. Enhancement limited to the geniculate ganglion, labyrinthine, and proximal tympanic facial nerve more often showed a complete return of facial function [12].

## Conclusions

A neoplastic etiology should be considered as a cause of FNP, particularly in cases where idiopathic FNP persists longer than 3 months, and a thorough work up is warranted in such cases. Work-up in FNP persisting over 3 months should include high-resolution MRI imaging and, as perilesional biopsies might be inadequate for rare cases like ours, consideration of a direct nerve biopsy to establish the right diagnosis.

## Abbreviations

BP: Bell´s palsy
CDDP: Cisplatin
CT: Computed tomography
EEG: Electroencephalography
FNP: Facial nerve palsy
MAC: Microcystic adnexal carcinoma
MRI: Magnet resonance tomography
PET-CT: 18-Fluoro-deoxyglucose-Positron-Emission-Tomography/ Computed Tomography (PET-CT)
SEPs: Somatosensory-evoked potentials
VZV: Varicella Zoster virus
